# Variability of computed tomography angiography coverage of lung parenchyma in acute stroke

**DOI:** 10.1186/s42466-021-00109-0

**Published:** 2021-03-02

**Authors:** Johannes A. R. Pfaff, Bianka Füssel, Marcial E. Harlan, Alexander Hubert, Martin Bendszus

**Affiliations:** Department of Neuroradiology, Heidelberg University Hospital, Im Neuenheimer Feld 400, 69120 Heidelberg, Germany

**Keywords:** Stroke, Thrombectomy, COVID-19, Computed tomography angiography, Lung

## Abstract

**Background:**

Computed tomography angiography (CTA) of the head and neck during acute ischemic stroke (AIS) usually includes visualization of lung apices. The possibility to evaluate for pulmonary changes, e.g. peripheral ground-glass and consolidative opacities suggestive of coronavirus disease 2019 (COVID-19)–related pneumonia, depends on the area of the lung covered by CTA.

**Methods:**

We performed an analysis of a real-world scenario assessing the variability of lung coverage on CTA in patients presenting with AIS to a comprehensive stroke center (CSC) or to one of eight primary stroke centers (PSC) within a teleradiological network covered by the comprehensive stroke center in 2019.

**Results:**

Our final analysis included *n* = 940 CTA, and in *n* = 573 (61%) merely lung apices were covered. In 19/940 (2%) of patients no lung tissue was covered by CTA. CTA scanning protocols in the CSC began significantly more frequently at the level of the ascending aorta (CSC: *n* = 180 (38.2%), PSC: *n* = 127 (27.1%), *p*-value < 0.001) and the aortic arch (CSC: *n* = 140 (29.7%), PSC: *n* = 83 (17.7%), *p*-value < 0.001), and by this covered less frequently the lower lobes compared to CTA acquired in one of the PSC.

**Conclusions:**

In our pre-COVID-19 pandemic representative stroke patient cohort, CTA for AIS covered most often only lung apices. In 37% of the patients CTA visualized at least parts of the lower lobes, the lingula or the middle lobe allowing for a more extensive assessment of the lungs.

## Background

Computed tomography angiography (CTA) of the head and neck as part of emergency imaging for acute ischemic stroke (AIS) is recommended for detection of a large vessel occlusion (LVO) and evaluation for mechanical thrombectomy [1]. Some authors advocate performing CTA and non-contrast-enhanced computed tomography (NCCT) together as an initial assessment for all AIS patients presenting within 24 h of last known well [2]. The measure is intended to improve LVO detection, increased the mechanical thrombectomy treatment population, hasten the intervention, and improved outcome among LVO patients.

Acquisition of CTA usually includes visualization of the upper thorax. This includes the lung apices and allows for evaluation for pulmonary opacifications. Assessment of lung tissue has gained more attention since a cluster of pneumonia cases in Wuhan, China marked the first patients infected with what is now known as severe acute respiratory syndrome coronavirus 2 (SARS-CoV-2) [3]. Based on positive reverse-transcription polymerase chain reaction (RT-PCR) test results, chest CT had a high sensitivity of 97% (95% confidence interval: 95–98%) for the diagnosis of coronavirus disease 2019 (COVID-19) in a Chinese population [4]. The high sensitivity of chest-CT for diagnosis of COVID-19 led the authors to conclude that chest-CT may be considered as a primary tool for detection of COVID-19 cases in epidemic areas [4]. In a subsequent study, the observations made with chest-CT could to some extend be reproduced in a smaller cohort in New York, USA, in patients who received a CTA for AIS, and thus, CTA has been advocated as an accurate screening tool for COVID-19 [5].

Since the studies that advocate chest-CT and CTA as a screening tool for COVID-19 came from regions with a local high rate of spread of the infection at the time of the conduct of the respective studies, transferability of the observations to other regions with lower incidences should be done with caution. Additionally, as pulmonary changes in patients with COVID-19 are more common in lower lobes, compared to the upper lobes [6], the reliability of CTA in the detection of pulmonary changes in patients with COVID-19 is limited. The extent to which lung tissue is covered may vary depending on the field of view of the CTA. The purpose of this study was to determine the area of lung parenchyma usually covered by a CTA in patients presenting with AIS.

## Methods

The data that support the findings of this study are available from the corresponding author upon reasonable request. This study was approved by the local ethics committee (Ethikkommission der Medizinischen Fakultät Heidelberg No: S-191/2020). Informed consent was waived.

For this retrospective study we included patients presenting to a university-based comprehensive stroke center or to one of eight primary stroke centers within a teleradiological network covered by the comprehensive stroke center and received CTA due to a suspected AIS. AIS was defined as hospital presentation for an acute neurological deficit that prompted CTA imaging within 24 h of symptom onset after consultation with the treating neurologist on site, respectively tele-neurologist. CTAs were included by identification of CTA examinations in the radiological information and workflow management system (Centricity™ RIS-i, General Electric Company, Boston, USA). In accordance with the preplanned statistical analysis permissible CTAs in the total cohort were limited to 50% to each group to avoid an excessive influence of CTAs performed either at the comprehensive stroke center or the hospitals within the teleradiology network. Every primary stroke center within the teleradiology network contributed at least *n* = 20 consecutive CTA examinations to the total cohort. Due to a higher patient volume, consecutive CTAs were performed within a shorter time period in the comprehensive stroke center, i.e. between January 1st, 2019, and April 13th 2019. CTAs in the teleradiological network were performed between January 1st, 2019, and July 21st, 2019. The scanning protocols in the comprehensive stroke center and the teleradiology network were standardized single-phase CTA aiming to begin at the level of the aortic arch. CTA studies were performed on commercially available CT scanners: comprehensive stroke center: Somatom Definition AS 64-Slice CT, Siemens Healthineers, Germany; primary stroke centers: 3 x Somatom Emotion 16-Slice CT, 1 x Somatom Scope Power 32-Slice CT, 1 x Somatom Sensation 40-Slice CT, 1 x Definition AS 20-Slice CT (all Siemens Healthineers), Aquilion Prime 80-Slice CT (Canon Medical Systems Corporation, Japan), MX16^Evo^ 16-Slice CT (Philips, The Netherlands).

**Fig. 1:**
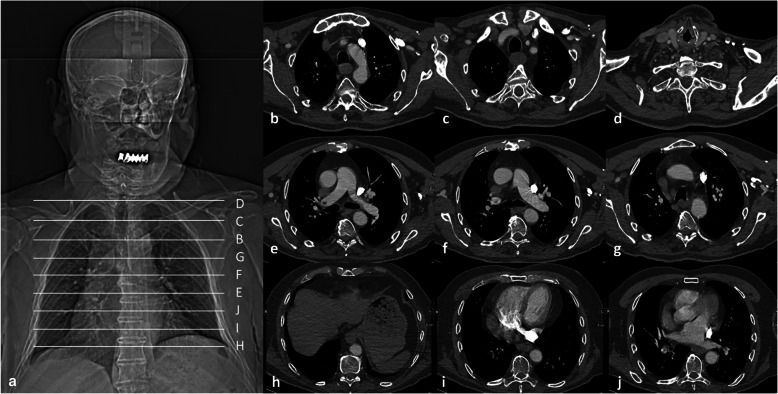
Examples of anatomical landmarks on axial CT angiography source images. **a** Shows a topogram of a CT angiography with reference lines corresponding to anatomical landmarks depicted in (**b**-**j**). Axial CT angiography images at the level of the aortic arch (**b**), supra-aortic vessels (**c**), cervical arteries (**d**), main pulmonary artery (**e**), carina (**f**) and ascending aorta (above the carina (**g**). The diaphragm and abdominal organs are shown in (**h**), the ventricles and left atrium in (**i**) and (**j**). Note: Depending on the level of the most caudal axial image the following lung segments are usually, at least partially, covered by CT angiography: B = right: 1–3, left: 1 + 2 and 3; C = right: 1 and 2, left: 1 + 2; D = no lung tissue; E = right: 1–3 and 6, left: 1 + 2, 3, 4 and 6; F = right: 1–3 and 6, left: 1 + 2, 3 and 6; G = right: 1–3, left: 1 + 2, and 3; H = right: 1–10, left: 1–10; I = right: 1–10, left: 1–10; J = right: 1–6, left: 1–6, and 8

The images were evaluated by a trained medical student (BF) and a board-certified radiologist subspecialized in neuroradiology (JARP) blinded to the site performing the CTA. CTAs were evaluated according to the (at least partial) presence of anatomical landmarks on the most caudal axial CTA source image (Fig. 1). In case of disagreement, consensus rating was reached with a second board-certified radiologist (AH).

Statistical analysis was performed by using SPSS Statistics (21.0.0.0; IBM, Armonk, NY). A two-sided level of significance with a *P* value of less than or equal to .05 was considered to indicate a significant difference.

## Results

Our final analysis included *n* = 940 CTA, i.e. *n* = 471 (50.1%) CTAs performed at the comprehensive stroke center, and *n* = 469 (49.9%) CTAs performed at one of the eight primary stroke centers. Mean patient age was 72 (SD: 14) years, and *n* = 448 (47.7%) CTAs were performed in male patients. Regarding age and sex, no difference was observed for patients who received CTA in the comprehensive stroke center vs. in a primary stroke center (Table 1).

**Table 1:** Demographic characteristics of patients who received CTA

	Overall	CSC	PSC	*p*-value
n (%)	940 (100)	471 (50.1)	469 (49.9)	
**Demographic Characteristics**				
Age, mean (SD), y	72 (14)	72 (15)	73 (14)	.296*
Sex				
Men	448 (47.7)	228 (48.4)	220 (46.9)	.648†
Women	492 (52.3)	243 (51.6)	249 (53.1)	

* Mann-Whitney-U test; † Fisher’s exact test, two sided; *CSC* Comprehensive stroke center, *PSC* Primary stroke center

In the overall patient population, most often the aortic arch (*n* = 223 (23.7%)) or the ascending aorta above the carina (*n* = 307 (32.7%) could be identified on the most caudal CTA image (Table 2). CTAs covered the caudal third of the thorax, i.e. beginning at the level of the left atrium or even below, in *n* = 132 (14.1%) patients.

**Table 2:** Distribution of anatomical landmark covered by CTA

	Overall	CSC	PSC	*p*-value	
n (%)	940 (100)	471 (50.1)	469 (49.9)		
**Anatomical landmark on the most caudal axial CTA source image**					
No lung parenchyma	19 (2)	4 (0.8)	15 (3.2)	< 0.001*	.01†
Supra-aortic vessels	43 (4.6)	18 (3.8)	25 (5.3)		.268†
Aortic arc	223 (23.7)	140 (29.7)	83 (17.7)		**< 0.001†**
Ascending aorta	307 (32.7)	180 (38.2)	127 (27.1)		**< 0.001†**
Carina	60 (6.4)	30 (6.4)	30 (6.4)		.986†
Pulmonary artery	156 (16.6)	64 (13.6)	92 (19.6)		.013†
Aortic valve or left atrium	27 (2.9)	10 (2.1)	17 (3.6)		.168†
Right or left ventricle	97 (10.3)	23 (4.9)	74 (15.8)		**< 0.001†**
Diaphragm or abdominal organs	8 (0.9)	2 (0.4)	6 (1.3)		.153†

* χ2 test, two sided, *p*-values with a level of significance < 0.05; † χ2 test, two sided; *p*-values following Bonferroni-Correction with a level of significance < 0.003; *CTA* Computed tomography angiography, *CSC* Comprehensive stroke center, *PSC* Primary stroke center; Significant *p*-values are printed in bold

CTAs in the CSC began significantly more frequently at the level of the ascending aorta (CSC: *n* = 180 (38.2%), PSC: *n* = 127 (27.1%), *p*-value < 0.001) and the aortic arch (CSC: *n* = 140 (29.7%), PSC: *n* = 83 (17.7%), *p*-value < 0.001). CTAs beginning at the level of the ventricles were more frequent in the PSC (CSC: *n* = 23 (4.9%), PSC: *n* = 74 (15.8%), *p*-value: < 0.001).

## Discussion

The main purpose of CT angiography (CTA) in patients with symptoms of an acute ischemic stroke is to visualize the vessels supplying the brain. CTA is intended to determine whether a vessel is occluded or stenotic and if so, in accordance with current guidelines, qualifies the patient for further endovascular treatment. The surrounding soft tissue, especially the lung apices, were previously merely included in the CTA imaging and received less attention in the context of acute stroke care. In this study, we demonstrated that in 61% of the overall patient population only lung apices and in 2% no lung tissue was covered by CTA. In 37% of the patients in this study, CTA covered segments of lingula, the middle lobe and the lower lobes. The latter finding was more frequent in patients who received CTA in a primary stroke center.

Assessment of lung tissue covered by CTA gained attention in the last month, as incidental pulmonary changes, i.e. peripheral ground-glass and consolidative opacities suggestive of coronavirus disease 2019 (COVID-19)–related pneumonia, have been reported in patients receiving CTA during stroke imaging [5, 7]. Esenwa and colleagues [5] reported a sensitivity of 0.67, specificity of 0.93, positive predictive value of 0.19, negative predictive value of 0.99, and accuracy of 0.92 of apical lung assessment on CTA for the diagnosis of COVID-19 when applying a categorical CT assessment scheme for patients suspected of having COVID-19 [8], and consider CTA an accurate screening tool for COVID-19. Esenwa et al. accurately describe that diagnostic accuracy is affected by local COVID-19 prevalence, and the results need to be used with caution in places with low prevalence.

Furthermore, identification of pulmonary changes suggestive for COVID-19 depends on timing of CT imaging in relation to symptom onset of COVID-19. Wang et al. report that temporal pulmonary changes of peak during illness days 6–11 of COVID-19 [9]. Additionally, pulmonary changes are more common in lower lobes (right: 84.3%, left: 83.4%), compared to the upper lobes (right: 66.2%, left: 68.75%) [6]. These circumstances limit the reliability of CTA in the detection of pulmonary changes in patients with COVID-19, and might even explain the lower sensitivity of CTA [5] compared to chest-CT [4]. In our study in a pre-COVID-19 real world scenario population, solely the upper lobes were covered in 71.7% of the CTA acquired in the comprehensive stroke center, and in 50.1% of the CTA acquired in primary stroke centers. In the study by Esenwa et al., CTA protocol included lung fields beginning at the level of the aortic arch. Thereby, not only ground-glass and consolidative opacities suggestive of COVID-19–related pneumonia, but also any kind of pulmonary changes (e.g. metastasis, atelectasis) in the lower lobes, the lingula or the middle lobe could have been missed.

In the eye of the COVID-19 pandemic, one might be inclined to extend the CTA scanning protocol caudally in order to cover more lung tissue and obtain a higher probability to identify pulmonary changes. This approach might be justified in cases where there is a high probability of pulmonary changes. However, starting CTA at a lower thoracic level will increase radiation exposure and thereby increase the risk of developing cancer, especially of radiosensitive organs such as the breast [10]. Additionally, an extension of the scanning protocol at the CTA does not necessarily have to lead to a higher probability of identifying patients with COVID-19. This has to do with the above-mentioned overestimation of the sensitivity of chest CT in the detection of COVID-19 [11]. For example, among passengers from the cruise ship *Diamond Princess*, i.e. representing an environmentally homogeneous cohort, *n* = 104 testet positive for COVID-19 (RT-PCR testing) and only *n* = 41 (54%) of which had lung opacities on chest-CT [12]. Correspondingly, the Fleischner Society recommend that imaging is not routinely indicated as a screening test for COVID-19 in asymptomatic individuals [13]. This underlines that CT is not the standard for the diagnosis of COVID-19. Imaging findings help to suggest the diagnosis of COVID-19 in the appropriate setting, however, it remains crucial to correlate findings from chest CT or CTA with clinical presentation, epidemiologic history, and RT-PCR test results.

Possible, voluntary changes to the CTA scanning protocol in light of the COVID-19 pandemic were considered and incorporated into the study design and led to choosing January to July 2019 as the study period. For this reason, our results are limited to report the variability of lung coverage by CTA in a general patient population, but not whether scanning protocols of CTAs have changed due to the COVID-19 pandemic. However, as the primary role of CTA of the head and neck for acute stroke imaging is to assess vascular pathologies, our scanning protocol remained unchanged. The reason why, despite a specified scanning protocol, variabilities occurred in the acquisition levels of CTA is unclear. Possible influencing factors might be (1) that during the planning of the CTA on a topogram the aortic arch cannot always be identified unequivocally, and accordingly, to be on the safe side, more was recorded from the thorax, (2) the training of the respective staff and (3) the frequency of the examination in the respective centers.

## Conclusion

In our pre-COVID-19 pandemic patient cohort, CTA for acute ischemic stroke covered most often only lung apices, but included at least parts of the lower lobes, the lingula or the middle lobe in 37% of the patients. With respect to the corona pandemic, the lung tissue covered by CTA may receive increased attention in order to raise suspicion of COVID-19 disease at an early stage for patients hospitalized for acute stroke. However, due to the its disadvantages, CTA should not be overestimated when screening for COVID-19, and the main focus of CTA should not shift from identifying vascular pathologies to screen for pulmonary changes.

## Data Availability

The data that support the findings of this study are available from the corresponding author upon reasonable request.

## Abbreviations

AIS: Acute ischemic stroke
COVID-19: Coronavirus disease 2019
CTA: Computed Tomography Angiography
MT: Mechanical thrombectomy
NCCT: Non-contrast-enhanced computed tomography of the head
RT-PCR: Reverse-transcription polymerase chain reaction
SARS-CoV-2: Severe acute respiratory syndrome coronavirus 2
